# Motor adaptation and immediate retention to overground gait-slip perturbation training in people with chronic stroke: an experimental trial with a comparison group

**DOI:** 10.3389/fspor.2023.1195773

**Published:** 2023-09-13

**Authors:** Tanvi Bhatt, Shamali Dusane, Rachana Gangwani, Shuaijie Wang, Lakshmi Kannan

**Affiliations:** ^1^ Department of Physical Therapy, College of Applied Health Sciences, University of Illinois, Chicago, IL, United States; ^2^ Ph.D. program in Rehabilitation Sciences, Department of Physical Therapy, College of Applied Health Sciences, University of Illinois, Chicago, IL, United States; ^3^ MS program in Rehabilitation Sciences, Department of Physical Therapy, College of Applied Health Sciences, University of Illinois, Chicago, IL, United States

**Keywords:** stroke, falls, stability, adaptation, reactive balance

## Abstract

**Background:**

Perturbation-based training has shown to be effective in reducing fall-risk in people with chronic stroke (PwCS). However, most evidence comes from treadmill-based stance studies, with a lack of research focusing on training overground perturbed walking and exploring the relative contributions of the paretic and non-paretic limbs. This study thus examined whether PwCS could acquire motor adaptation and demonstrate immediate retention of fall-resisting skills following bilateral overground gait-slip perturbation training.

**Methods:**

65 PwCS were randomly assigned to either (i) a training group, that received blocks of eight non-paretic (NP-S1 to NP-S8) and paretic (P-S1 to P-S8) overground slips during walking followed by a mixed block (seven non-paretic and paretic slips each interspersed with unperturbed walking trials) (NP-S9/P-S9 to NP-S15/P-S15) or (ii) a control group, that received a single non-paretic and paretic slip in random order. The assessor and training personnel were not blinded. Immediate retention was tested for the training group after a 30-minute rest break. Primary outcomes included laboratory-induced slip outcomes (falls and balance loss) and center of mass (CoM) state stability. Secondary outcomes to understand kinematic contributors to stability included recovery strategies, limb kinematics, slipping kinematics, and recovery stride length.

**Results:**

PwCS within the training group showed reduced falls (*p* < 0.01) and improved post-slip stability (*p* < 0.01) from the first trial to the last trial of both paretic and non-paretic slip blocks (S1 vs. S8). During the mixed block training, there was no further improvement in stability and slipping kinematics (S9 vs. S15) (*p* > 0.01). On comparing the first and last training trial (S1 vs. S15), post-slip stability improved on both non-paretic and paretic slips, however, pre-slip stability improved only on the non-paretic slip (*p* < 0.01). On the retention trials, the training group had fewer falls and greater post-slip stability than the control group on both non-paretic and paretic slips (*p* < 0.01). Post-slip stability on the paretic slip was lower than that on the non-paretic slip for both groups on retention trials (*p* < 0.01).

**Conclusion:**

PwCS can reduce laboratory-induced slip falls and backward balance loss outcomes by adapting their post-slip CoM state stability after bilateral overground gait-slip perturbation training. Such reactive adaptations were better acquired and retained post-training in PwCS especially on the non-paretic slips than paretic slips, suggesting a need for higher dosage for paretic slips.

**Clinical registry number:**

NCT03205527

## Introduction

Stroke is the third leading cause of disability in the United States, affecting nearly 800,000 people annually (1). Approximately, 50% of people with chronic stroke (PwCS) experience residual hemiparesis, predisposing them to falls (1). Every year about 40%–70% of community-dwelling PwCS experience detrimental falls during walking (2–4), resulting in a four-fold increased risk of hip fractures, especially on the hemiparetic side (5). Thus, falls, fall-related injuries, and the subsequent reduction in physical activity levels and deconditioning pose a major health and economic burden among PwCS (6). Given the detrimental nature of falls, it is imperative to design effective training paradigms that improve postural stability during walking and reduce fall risk in PwCS.

Conventional physical therapy alone (7, 8), and in combination with exercise-based protocols, has been shown to improve balance and mobility in PwCS (9, 10), however, there is limited evidence that such protocols reduce real life falls (11). Such limited generalization might be attributed to the lack of task-specificity, insufficient dosage (less training intensity or frequency of sessions), and reduced adherence and compliance at home or in community settings. The lack of sufficient task-specific practice might hinder neural reorganization required for functional recovery and long-term gains (12).

An emerging task-specific paradigm for fall prevention is perturbation-based training (13, 14). Such training involves repeated exposure to perturbations that alter the relationship between an individual's center of mass (CoM) and base of support (BOS), resulting in a loss of balance situation similar to that in real life. Recovery from balance loss requires effective reactive balance strategies, such as the “fixed-support” or “feet-in-place” strategy, usually seen in response to small perturbations where postural responses are elicited to restore the displaced CoM within the BOS (feet) or the “change-in-support” strategy in response to larger perturbations where the BOS is altered usually by taking a compensatory step to re-establish the CoM within the BOS (15). Perturbation training has been shown to improve control of these reactive balance strategies resulting in improved CoM stability and reduced balance loss/fall outcomes (13, 16–18). Overground slip perturbation training in community-dwelling older adults demonstrated rapid adaptive improvements in reactive CoM state stability and reduced laboratory falls from 49% on the first slip to no falls on the 24th slip (18). Such adaptive effects have been shown to generalize to different tasks and contexts within the laboratory setting and can be retained for up to a year (18–20). Most importantly, such training reduced the annual risk of real life falls by 50%, indicative of long-term generalization and retention of acquired fall-resisting skills (18). Further, the “first trial effect” characterized by acquired improvement following exposure to a single slip has also resulted in improved laboratory fall rates and stability control after 6, 9, and 12 months (19). These findings demonstrate the magnitude of adaptation and profoundness of the overground slip training paradigm in older adults.

Similarly, perturbation training has been utilized for fall prevention in PwCS. Preliminary evidence in PwCS has demonstrated that repeated exposure to both small (pull-push) (20–22) and large (treadmill-based) magnitude perturbations in standing result in improved recovery stepping responses and postural stability (23–26). These findings are indicative of the preserved ability of PwCS to undergo reactive adaptation during standing (27, 28). However, only a few studies have examined perturbation training effects during gait in people with stroke (35–38). Two case studies performed by Matjačić et al. (29) and Olensek et al. (30) used a balance-assessment robot to deliver random perturbations to the participant's pelvis while walking on an instrumented treadmill. After such gait-perturbation training, PwCS successfully counteracted high and low amplitude perturbations during treadmill walking and demonstrated increased successful paretic cross-stepping, i.e., there were no collisions with the non-paretic limb while walking post-training (29). In a related study by Handelzalts et al. (31), 34 individuals with sub-acute stroke underwent 12 sessions of unexpected perturbations during standing and treadmill walking. Participants demonstrated improved reactive balance as indicated by a higher multiple-stepping threshold i.e., multiple-stepping responses elicited at a higher intensity post-training than pre-training, and an increase in participants' self-reported balance confidence post-training.

Although these studies highlight the efficacy of perturbation training paradigms in individuals with stroke, they do not report on within-session acquisition changes in postural stability or recovery stepping, nor elaborated on the factors responsible for adaptation. Further, while motorized perturbations possibly entrain fall-resisting skills, they are mostly delivered during split-belt treadmill walking which provide unilateral slips, however, might not mimic real-life slips, as the slip intensity are preprogrammed and rarely affected by the participant's gait behavior and reactive action. On the contrary, overground perturbations can simulate real-life slips along with providing distinct unilateral perturbations allowing us to examine reactive responses following non-paretic and paretic slips. Studies inducing novel, unexpected slip perturbations in PwCS during overground walking have demonstrated comparable fall risk following both non-paretic and paretic slips (15, 32–34). Moreover, real life slips could occur under either limb, thereby necessitating contributions from both limbs in effective recovery stepping and weight bearing for fall prevention. The paretic limb has been associated with an impaired ability to initiate a successful recovery stepping response when the non-paretic limb is the slipping limb and is shown to have poor vertical stance limb support when it is the slipping limb (32). This highlights the need for bilateral perturbation training during overground walking to facilitate the initiation and appropriate execution of recovery response from the paretic limb during a non-paretic slip and train the paretic limb to effectively weight bear and control the slip when it is the slipping limb (32).

Thus, the purpose of this study was to determine whether PwCS could acquire and retain motor adaptation changes following a block-and-mixed perturbation training protocol applied to both paretic and non-paretic limbs. We hypothesized that PwCS within the training group would demonstrate a reduced incidence of laboratory-induced slip falls and significant proactive (pre-slip) and reactive (post-slip) adaptations in CoM stability control following block training involving repeated slip trials on the non-paretic limb and subsequently the paretic limb. Such acquired adaptations would be maintained during the mixed block training during which participants would be exposed to a random sequence of paretic and non-paretic slips mixed with unperturbed natural walking trials. Further, adaptive changes in stability would be associated with changes in kinematic parameters. We also hypothesized that the training group would demonstrate immediate within group retention (after a 30-minute rest break) of the acquired adaptations resulting in significantly lower falls and greater stability on the re-test paretic or non-paretic slips compared to the last training slips, respectively. Lastly, we hypothesized that the training group would demonstrate effects of the slip intervention on falls and stability as compared to the control group (who received no prior slip exposure).

## Methods

### Participants

Sixty-five community-dwelling PwCS (>6 months post-cortical stroke as confirmed by their physician) who were able to ambulate independently with or without an assistive device were included. During the in-person screening, participants were excluded in the presence of cognitive impairments (score of ≤26/30 on Montreal Cognitive Assessment Scale, MOCA), speech impairments (aphasia score of ≥71/100 on Mississippi Aphasia Screening Test), poor bone density (T score <−2 on the heel ultrasound) or any other self-reported neurological, musculoskeletal, or cardiovascular conditions other than underlying effects from the stroke such as traumatic brain injury, spinal cord injury, Parkinson's disease, peripheral neuropathy, osteoarthritis, rheumatoid arthritis, acute or chronic congestive heart failure, chronic obstructive or restrictive lung disease. All participants provided written informed consent as approved by the institutional review board of the University of Illinois at Chicago before their enrollment. The assessor and training personnel were not blinded. The participants were blinded to the study aims.

### Baseline assessment during in-person screening

**Figure 1:**
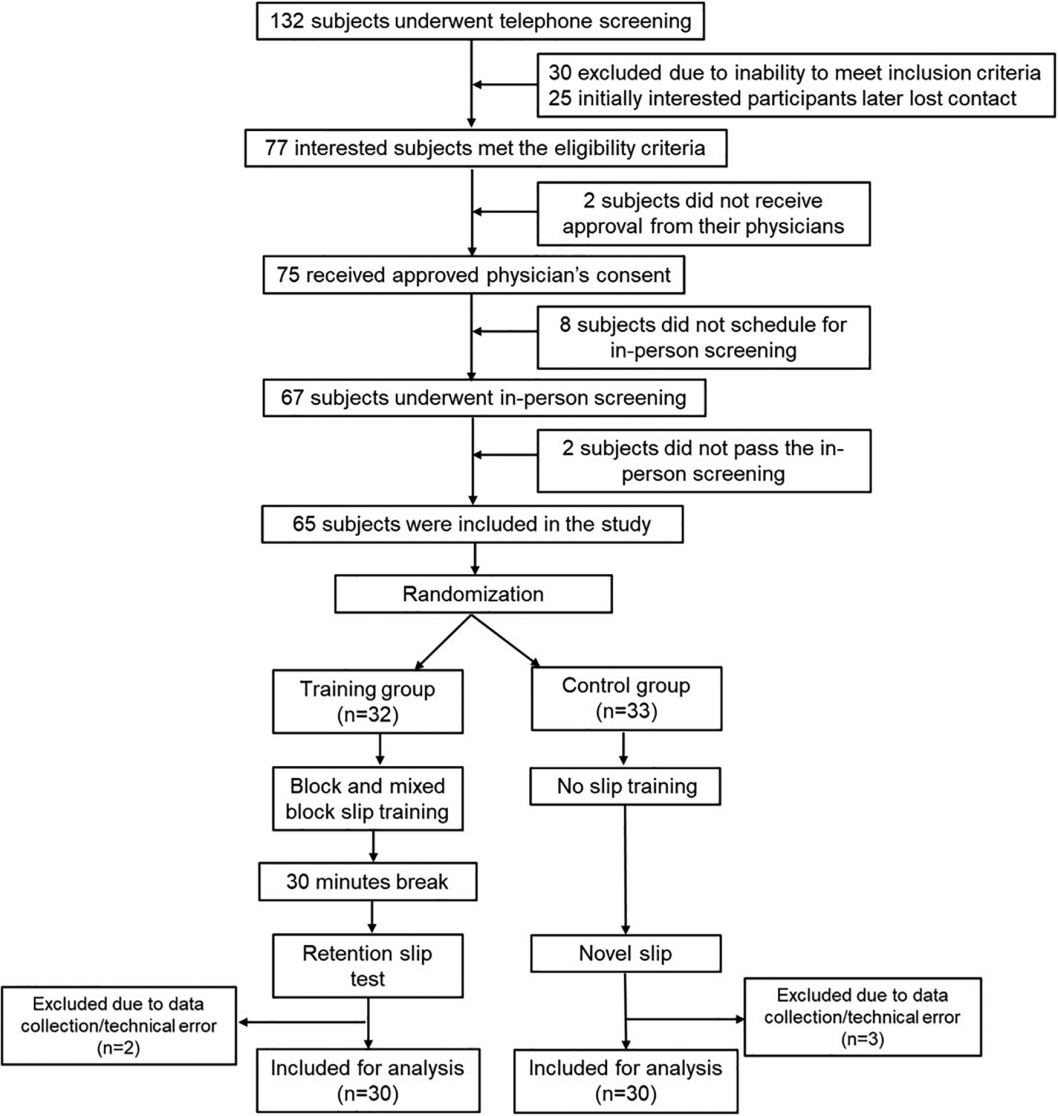
Study flowchart: the data presented in this study is a part of a larger ongoing clinical trial (Grant Number: 1R01HD088543-01A1). The CONSORT flowchart presented pertains only to the data presented in this manuscript.

Baseline assessment included clinical outcome measures such as the Berg Balance Scale (BBS) (35, 36), Timed Up and Go test (TUG) (37, 38), 10 meter walk test (10MWT) (39–41), chronicity of stroke, and severity of motor impairment using the Chedoke-McMaster Stroke Assessment scale (CMSA) (42, 43). Following the initial screening and baseline assessment, participants were randomized into training or control groups using the RAND function in Microsoft Excel (Figure 1).

### Overground gait-slip protocol

**Figure 2:**
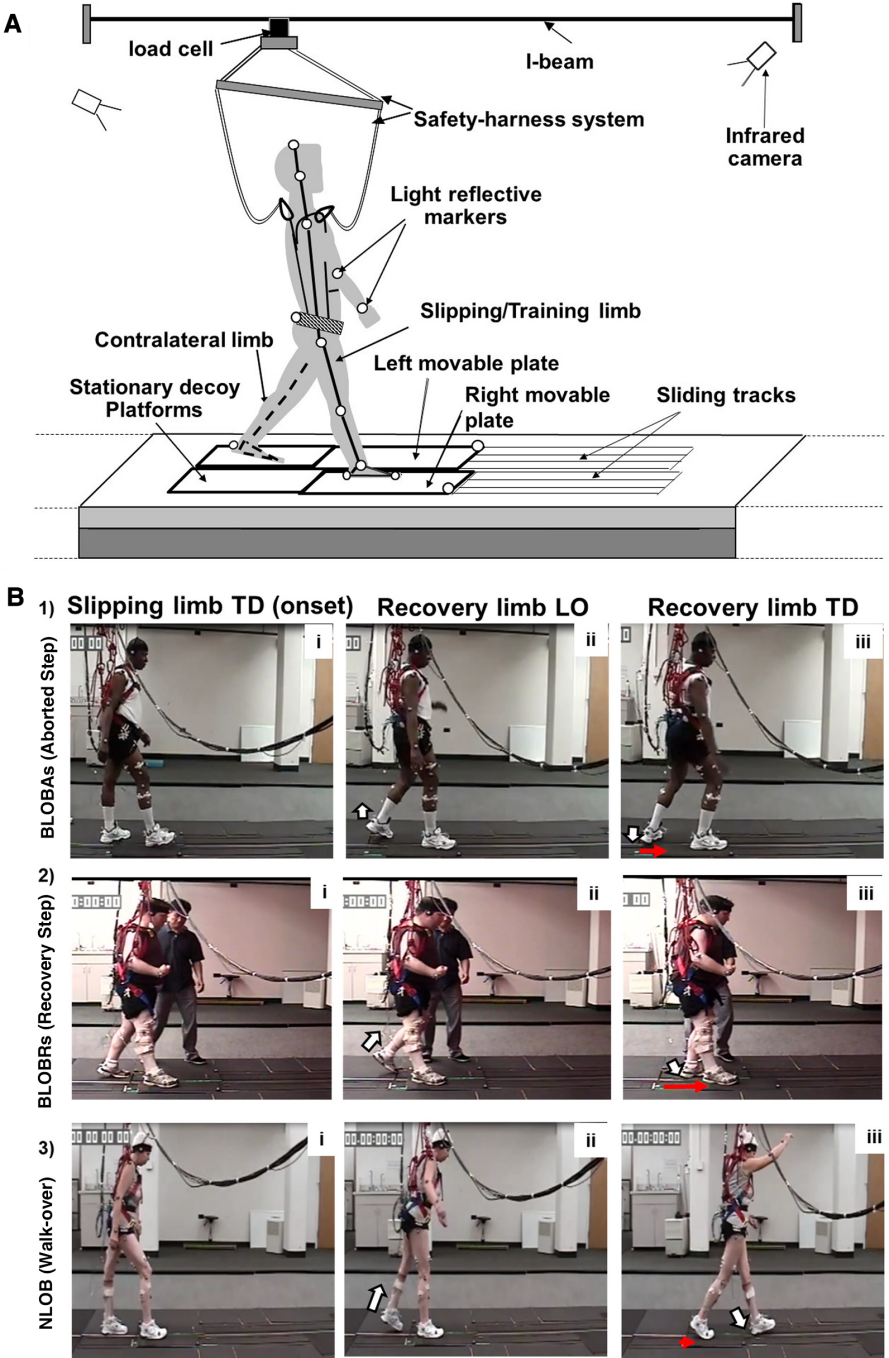
(**A**) Schematic diagram of the experimental setup. The 8-meter-long overground walkway with two low-friction, nonmotorized moveable top plates (right and left) mounted on a frame with linear bearings. Both moveable platforms were embedded in the walkway and surrounded by stationary decoy platforms. Passive-reflective markers on the body segments of the participant and the platform are indicated by unfilled circles. The participant wore a safety harness attached to the load cell mounted on an overhead trolley on a track over the walkway (i-beam). (**B**) Schematic showing backward loss of balance (BLOBAs or BLOBRs) (with an aborted and recovery step) vs. no loss of balance (NLOB) post-slip outcomes. Three sections of the figure indicate the key instances of (i) slipping limb touchdown (TD) at slip onset, (ii) post-slip recovery limb liftoff (LO), and (iii) post-slip recovery limb TD. The white arrow indicates the LO and TD of the recovery limb (shown in panels ii and iii), while the red arrow indicates the movement of the slipping platform at touchdown of the recovery limb (shown in panel iii). (**B1**) shows BLOBAs following non-paretic slip wherein unloading of the limb is followed by sudden loading of the recovery limb without complete toe clearance. (**B2**) shows BLOBRs following paretic slip wherein forward stepping recovery limb lands posterior to the slipping limb. (**B3**) shows NLOB following non-paretic slip wherein the heel marker of the recovery foot is anterior to the heel marker of the slipping foot in the anteroposterior direction at the recovery TD with minimal forward displacement of the slipping heel, resulting in a walk-over.

Figure 2A shows the schematic of the experimental setup. Participants were secured in a firmly fitting safety harness to prevent them from injuring themselves in the event of a fall. The harness system included a full-body safety harness, rope wires, and an S-type load cell. The harness was connected to the load cell mounted on an overhead trolley on a track over the walkway with shock-absorbing ropes (see data collection section below). The load cell was calibrated with a few known weights within the region of bodyweight and scaled to Newtons using a least square regression technique; the calculated beta values were used to convert voltage data into weight.

Once the set-up was complete, participants were asked to walk along an 7-meter instrumented walkway to familiarize them with the new laboratory environment. Baseline walking trials were collected at the participant's preferred speed after the familiarization. Participants' gait speed was not imposed to allow participants to walk at their preferred speed similar to that in their daily life.

We further confirmed that slips were induced at the individual's preferred gait speed, by comparing the walking gait speed of 5 unperturbed baseline trials with their speed during the 10-meter walk test (instructed to walk at preferred speed) and found no significant differences between the two speeds (*p* = 0.31). After baseline walking trials and before beginning the repeated slip perturbation training, participants were instructed that they may experience a slip under either limb without any warning and to recover their balance and continue walking. Slips were induced by a pair of low-friction moveable platforms that were embedded in the 7-m walkway and surrounded by stationary decoy platforms (42). Each of the moveable platforms was 65 cm × 30 cm and mounted on a 2.5 m supporting metal frame. Two individual force plates were installed underneath each frame to measure ground reaction forces. On the trials that were designated as slip trials, the experimenter would enable the trigger button in the data collection software that would then automatically release the moveable plate from a locked position once the detected ground reaction force under the slipping limb exceeded a preset threshold of 20 N. Once released the moveable platform was free to slide up to a maximum slip distance of 45 cm, while the other platform remained locked (stationary) leading to a single-foot slip (Figure 2A). The exact location and the time of the slip trigger were not known to the participant. Participants starting line was adjusted without their knowledge (for example, when the participant was walking on the walkway facing the opposite direction) to ensure landing on the desired movable plate.

### Intervention protocol

**Figure 3:**
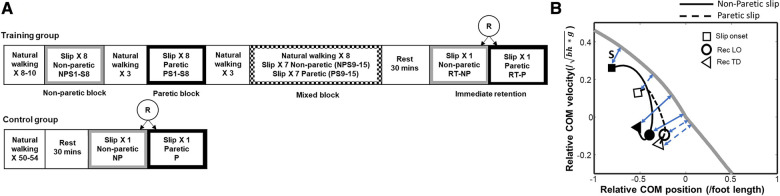
(**A**) Overground gait-slip training protocol used for people with chronic stroke (PwCS). R indicates randomization; NP indicates non-paretic; P indicates paretic; S in S1, S8, S9, S15 indicates slip and the following number indicates the trial number, (**B**) Center of mass (CoM) state trajectories for a non-paretic slip (solid line) and a paretic slip (dotted line) for a representative subject. The thick gray line represents the threshold for backward loss of balance (BLOB). The time course of the CoM state trajectory is from slip onset (indicated by open square), to recovery liftoff (Rec LO, indicated by circle), to recovery touchdown (Rec TD, indicated by triangle). A person whose CoM state is below the threshold after slip onset is likely to experience a BLOB. A CoM state above the threshold indicates a reduced likelihood of BLOB. Positive values of relative CoM position indicate that the CoM is anterior to the base of support (BOS), and negative values indicate that the CoM is posterior to the BOS. Positive values of the relative CoM velocity indicate that CoM is traveling forward faster than the BOS, and negative values indicate that it is traveling forward slower than BOS. The instantaneous stability (S) for a CoM state is calculated as the shortest perpendicular distance (blue double-headed arrow) between the BLOB threshold and the CoM state. The stability at the key time events (slip onset, rec LO and rec TD) for non-paretic slip are indicated by blue solid double-headed arrow, and the stability for paretic slip are indicated by blue dashed double-headed arrow. The more negative the stability measure is, the greater the likelihood of backward balance loss. Here, g is the acceleration due to gravity, and bh is the body height.

Following baseline walking, participants from the training group received a block of 8 consecutive non-paretic slips (NP-S1 to NP-S8) followed by a block of 8 consecutive paretic slips (P-S1 to P-S8) (Figure 3A). Participants were not given any explicit instructions to modify their walking. Our previous findings have demonstrated that paretic slips can be more challenging for PwCS, especially with an increased slip distance of 45 cm (44). Additionally, the presence of neuromuscular impairment and risk of injury on the paretic limb might compromise patient safety and result in poor tolerance with paretic slip training. Thus, the non-paretic slip block was given first to ensure patient safety and to allow for better learning. After block training, participants were then subjected to a mixed block consisting of 13 walking trials interspersed with slipping trials under non-paretic and paretic limbs in a pseudo-random order (NP-S9/P-S9 to NP-S15/P-S15). The sequence of walking and slip trials were kept the same for all participants in the training group. Post-training, participants were given a 30-minute rest break following which they were then randomly exposed to a single trial of each non-paretic (RT_NP) and paretic slip (RT-P) to determine immediate retention of training effects at individual preferred gait speeds. The research protocol design was planned to deliver block training to both limbs to facilitate the acquisition of adaptation followed by a mixed block of paretic and non-paretic slips and unperturbed walking trials. This was done to reduce reliance on anticipation and develop a steady state stability that could prevent balance loss or a fall upon encountering either a paretic or non-paretic slip. Such mixed block training (random training) following block training is also known to enhance retention (17, 18, 45).

### Control protocol

Participants assigned to the control group received an unexpected gait slip randomly under their non-paretic and paretic limbs following the baseline walking trials. They were not given baseline slips followed by a retest after 30 min as our previous studies have shown a single trial effect in older adults and we did not want to add this bias (46).

### Data collection

An 8-camera motion capture system (Motion Analysis Corporation, Santa Rosa, CA) was used to record full body kinematics using a set of 30 retro-reflective modified Helen Hayes markers. Kinematic data were sampled at 120 Hz and synchronized with the force plate [0R65-1000 (AMTI, Newton, MA)] and load cell data [BSS-1k (Transcell Technology Inc., Buffalo Grove, IL)], which were collected at 600 Hz. Reflective markers were placed on the movable platform to calculate slipping kinematics. The instances of liftoff (LO) and touchdown (TD) were determined from the vertical ground reaction forces using a custom-written Matlab program. The marker data collected for three participants were not complete (as markers fell off from the participants during the key slips trials of interest). Due to technical problems, the slip slider was not triggered for some of the key trials, (the novel slip on either of the blocks and/or the retention slip trial) for 5 of the participants. Hence, the data of these five participants were disregarded (three from the control group and two from the training group).

### Data analysis

#### Primary outcome measures

##### Slip outcome:

Outcomes of slip trials were categorized as a fall or a recovery depending upon the weight borne by the harness. Trials with more than 30% of the participant's body weight were categorized as falls while the remaining slip trials were categorized as recovery (47).

##### Pre- and post-slip center of mass (CoM) state stability:

The region of stability at given CoM locations is defined as the feasible range of CoM velocities that can be reduced to zero with respect to the BOS while still allowing the CoM to traverse within the BOS limits (48). The BOS is the distance in the anterior-posterior direction between leading toe and trailing heel during the double-support phase when both feet are in contact with the ground (49). Once the CoM state (instantaneous CoM position and velocity) travels outside the posterior boundary of the region of stability, a backward loss of balance (BLOB) would occur. Therefore, the CoM state stability was calculated as the shortest distance from the CoM state to the computational threshold against BLOB (posterior boundary) during slipping (50) and is expressed as a dimensionless measure. Figure 3B demonstrates a representative COM state trajectory from a non-paretic and paretic limb slip plotted against the computational threshold of backward balance loss. Both trials resulted in a BLOB with negative stability values. The CoM position was computed from 12 segment body representation (51), expressed relative to the most posterior BOS (i.e., slipping or trailing/recovery limb heel) and normalized by the participant's foot length. While CoM velocity was derived from CoM position, it was normalized by a dimensionless fraction of √g*h, where *g* is the acceleration due to gravity and *h* is the participant's height and expressed relative to the BOS. A posterior shift in the CoM position will move the CoM away from the BOS resulting in less positive values and if the CoM is outside the BOS it will result in negative values. Similarly, a slower-moving CoM compared to the BOS will result in negative CoM velocity and vice versa. A CoM state stability <0 indicates a high possibility of BLOB while CoM stability >0 indicates a low possibility of a BLOB. The CoM state stability was determined relative to the slipping side BOS (heel), at slip limb TD for pre-slip stability and recovery limb TD for post-slip stability (Figure 3B).

#### Secondary outcome measures

##### Recovery strategies

Recovery strategies following slip perturbations were identified as backward loss of balance (BLOB), defined as the need to execute a recovery stepping response, or as no loss of balance (NLOB) when recovery stepping was not needed, and the participants continued to maintain their regular walking pattern (Figure 2B). A BLOB was identified based on the landing location of the recovery foot. If the heel marker of the recovery foot was posterior to the heel marker of the slipping foot in the anteroposterior direction at the recovery TD, the trial would be classified as a BLOB, otherwise, it would be classified as an NLOB (17). A BLOB was associated with either an aborted (BLOBAs) or a recovery (BLOBRs) step. An aborted step was identified as unloading followed by sudden loading of the recovery limb without complete toe clearance (52, 53) [Figure 2B (1)], while a recovery step was identified as a forward step of the recovery limb which lands posterior to the slipping limb [Figure 2B (2)]. A NLOB was associated with either a walk-over (NLOBWO) or a skate-over adaptive strategy (NLOBSkO) [Figure 2B (3)]. Both walk-over and skate-over adaptive strategies involve the recovery limb landing anterior to the slipping limb. A walk-over strategy was identified when a participant performed a forward step with the recovery limb that landed anterior to the slipping limb, resembling a natural walking pattern during a slip trial, with a minimal forward slipping heel displacement (≤0.05 m) (13, 54). A skate-over strategy was identified when the participant performed a forward step with the recovery limb that landed anterior to the slipping limb but with a forward heel displacement of >0.05 m i.e., greater than that during a walk-over strategy (13, 54).

##### Slipping kinematics

Peak slip displacement: Peak slip displacement was calculated as the maximum displacement covered by the slipping foot heel marker, measured in meters (m), and maximum slip velocity was computed as the first-order derivative of slip displacement, measured in meter per second (m/s) between the pre-slip touchdown and post-slip lift off of the slipping limb.

Recovery stride length: Recovery stride length during gait-slip was calculated as the distance traveled by the metatarsal marker of the recovery limb from its lift-off to touchdown and was normalized by body height in meters.

##### Limb kinematics

Previous studies indicated that the trunk angle (55), ankle angle, and knee angle of the slipping limb (56) play a key role in balance control, hence, these angles were calculated at pre-slip TD and post-slip recovery step TD, respectively. The trunk angle was defined as the angle between the trunk segment (a straight-line joining midpoint of the shoulder marker and the midpoint of the hip marker) and a vertical line in the sagittal plane. The knee angle was defined as the angle between the shank and thigh segment in the sagittal plane. Ankle angle was defined as the angle between the foot and shank segment in the sagittal plane. The thigh segment was computed using markers on the greater trochanter and lateral condyle of the femur. The shank segment was computed using the lateral condyle of the femur and lateral malleolus of the ankle. The foot segment was computed using heel and toe markers.

#### Statistical analysis

An a-priori power analysis was conducted to detect the sample size using the G*Power version 3. Based on our pilot work in *older adults*, an estimated sample size of 35 participants in each group could detect a between-group difference in post-slip stability at touchdown with a power of >80% at a two-sided alpha level of 0.05. This sample size would also give us >80% power for an estimated difference of 40% in falls between the training and control group.

##### Adaptation to block and mixed training

Friedman's test using Chi-square statistics was used to determine the effect of overground gait-slip training on fall (fall vs. no fall) and balance loss outcomes (BLOB vs. NLOB) for the training group. Within the training group, the time-based effects of training were examined using planned comparisons for significant pairs using the Wilcoxon-signed rank test between the first and last trials of the non-paretic block (NP-S1 vs. NP-S8), paretic block (P-S1 vs. P-S8), and mixed block (NP-S9 vs. NP-S15, P-S9 vs. P-S15) and between the first and last non-paretic (NP-S1 vs. NP-S15) and paretic (P-S1 vs. P-S15) trials. Respective one-way repeated measures analysis of variance (ANOVA) was conducted involving only the slip trials to determine adaptation across trials in pre-slip and post-slip CoM state stability. To resolve the significant main effects, post-hoc planned comparisons using paired t-tests were conducted for the same comparisons done for the non-parametric tests mentioned above. Repeated measures ANOVAs were also performed for maximum heel displacement, heel velocity, and recovery stride length including selected trials (NP-S1, NP-S8, P-S1, P-S8, NP-S9, P-S9, NP-S15, and P-S15 and the retention trials—RT-NP, RT-P). An alpha correction was applied based on the number of planned comparisons performed resulting in a level of significance at a p-value of 0.01.

##### Kinematic predictors of stability

To explore the key factors that contribute to the variance in stability (or the gait stability adaptation) during repeated slip training, stepwise linear regression was conducted to examine the relationship between pre-slip stability at slipping foot touchdown and kinematic variables (gait speed, step length, trunk angle, ankle, and knee joint angles of the slipping limb at pre-slip TD) using all trials from the first (non-paretic) and second (paretic) repeated training blocks. To examine the contribution of slip intensity on post-slip CoM state stability, a univariate regression was done between post-slip stability and slip distance at its recovery step touchdown using the same trials. After that, another stepwise linear regression was conducted to explore which variable(s) could affect the slip distance among gait speed, step length, pre-slip stability, trunk angle, ankle angle, and knee angles of the slipping limb at pre-slip TD and recovery step touchdown. The variables for these analyses were chosen based on previous research that has identified potential contributing factors to changes in pre- and post-slip stability and slip kinematics. For each stepwise linear regression, all the potential contributors were inputted into the regression model, and any factor with a *p*-value over 0.05 was excluded by the model. A partial correlation was run to determine the relationship between individual independent variables with the respective dependent variable. Partial r square value was calculated using the equation (SSE(reduced)—SSE(full))/SSE(reduced) where SSE stands for the sum of squared estimate of errors, SSE(full) stands for the SSE based on all predictors selected by stepwise method and SSE(reduced) stands for the new SSE after removing one predictor.

##### Effects of immediate retention of acquired adaptation (within training group)

To examine the effects of within-group retention of acquired adaptation in the training group, planned comparisons between the last slip trial of the mixed block and the retention trial were performed for the non-paretic slips (NP-S15 vs. RT-NP), and the paretic slips (*P*-S15 vs. RT-P).

##### Effects of slip training (between groups)

The control group's novel slips would provide a between-group comparison for the retention trials of the training group to understand the effect of slip training. To determine the effect of slip training on fall incidence a 2 × 2 Generalized Estimating Equations model [group (training vs. control) vs. trial (NP vs. P)] was performed and significant effects, if any, were followed by Mann Whitney U tests for between-group comparisons (RT-NP vs. control-NP; RT-*P* vs. control-P) and Wilcoxon-signed rank tests for within-group comparisons (NP vs. P for training and control groups). Similarly, for pre-slip and post-slip CoM state stability, respective 2 × 2 repeated measures ANOVAs were performed. To resolve the main effects, planned independent (between) and paired t-tests (within) were conducted, with an alpha level of 0.01 (correcting for multiple comparisons).

To determine the impact of age, BMI, and gender on pre- and post-slip stability for immediate retention for both groups, an analysis of covariance (ANCOVA) was performed. All analyses were performed using SPSS version 24 keeping with an alpha level of 0.05 for overall analysis and corrected alpha levels as described above for planned comparisons.

## Results

Figure 1 shows the CONSORT flow diagram for this study showing participant numbers for recruitment, screening, and inclusion. A sample of 132 participants was screened of which 75 met inclusion criteria and had approved physician's consent. However, 8 participants failed to schedule their in-person screening and 2 participants failed the in-person screening (heel ultrasound T score >−2). Thus, following the initial screening and baseline assessment, a total of 65 participants were randomized with *n* = 32 in the training group and *n* = 33 in the control group (Figure 1). Of the sixty-five community-dwelling PwCS (>6 months post-cortical stroke as confirmed by their physician), 55% of participants had an ischemic stroke, 30% had a hemorrhagic stroke, and 15% with an unknown type of cortical stroke). Although 65 participants were enrolled, the total number of participants included for data analysis were *n* = 30 in each group (5 participants' data were excluded due to data collection/technical error). The demographic details of the included participants are presented in Table 1. There were no significant differences between groups at baseline (*p* > 0.05) (Table 1).

**Table 1:** Demographics and clinical outcome measures for study participants.

	Training group	Control group
	*n* = 30	*n* = 30
Age (years)	57.89 ± 8.49	61.17 ± 12.11
Height (meters)	1.72 ± 0.10	1.71 ± 0.11
Weight (kilograms)	81.46 ± 13.60	83.72 ± 16.98
Gender (Male/Female)	21/9	17/13
Severity of disability (Modified Rankin scale)	1.83 ± 0.58	2.10 ± 1.04
Chronicity of stroke (years)	10.30 ± 6.52	8.97 ± 4.98
Haemorrhagic/Ischemic	8/17	10/16
Impairment level		
CMSA (Leg)	5.07 ± 0.93	5.03 ± 1.14
CMSA (Foot)	3.60 ± 1.2	4.30 ± 1.53
Balance (BBS)	49.56 ± 4.9	47 ± 8.42
TUG (s)	19.70 ± 12.15	18.54 ± 15.23
Gait speed (m/s) (Average of 5 unperturbed trials)	0.74 ± 0.23	0.71 ± 0.34

CMSA, Chedoke-McMaster Stroke Assessment scale; BBS, Berg Balance Scale; TUG, Timed Up and Go test; s, Seconds, m/s, meter per second.

Statistical analysis showed no significant differences between groups at baseline (*p* > 0.05).

Our overground gait-slip protocol was well-tolerated by all the participants assigned to the training and control group. There were no adverse events or side effects reported following the intervention.

### Adaptation to block and mixed training

#### Slip outcomes

**Figure 4:**
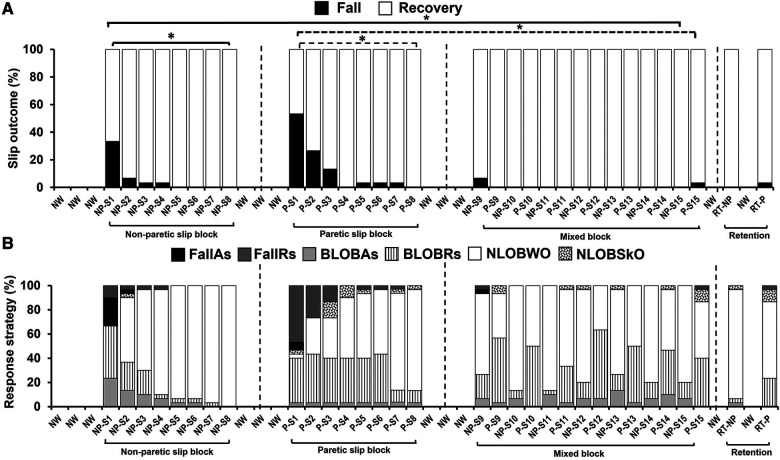
Percentage of (**A**) slip outcomes [falls and recovery (no falls)] and (**B**) response strategies employed by the training group on the non-paretic (NP), paretic (P) and retention (RT) slip trials [fall with aborted step (FallAs), fall with recovery step (FallRs), backward loss of balance with aborted step (BLOBAs), backward loss of balance with recovery step (BLOBRs), no loss of balance walkover (NLOBWO) and no loss of balance skate over (NLOBSkO)]. Natural walking trials are referred to as NW. Significant differences in fall percentage (*p* < 0.01) are indicated by *. Solid lines indicate differences in non-paretic slips between NP-S1 and NP-S8 and between NP-S1 and NP-S15 trials. Dotted lines indicate differences in paretic slips between P-S1 and P-S8 and between P-S1 and P-S15 trials.

Friedman's test showed a significant reduction in % of falls across trials [*X^2^* *=* *237.66, p* *<* *0.001*] which was associated with a reduction in balance loss and the emergence of the walkover and skate-over recovery strategies (Figures 4). Planned comparisons showed that there was a significant decrease in falls from NP-S1 (33%) to NP-S8 (no falls) (*Z* *=* *−3.16, p* *<* *0.01*) and from P-S1 (53%) to P-S8 (no falls) (*Z* *=* *−4, p* *<* *0.01*). However, there was no difference in the mixed block between the first and last trials on non-paretic [NP-S9 vs. NP-S15 (*Z* *=* *−1.41, p* *>* *0.01*)] and paretic limb [P-S9 vs. P-S15 (*Z* *=* *−1.0, p* *>* *0.01*)]. Lastly, a significant reduction in falls between the first and last training trials on both non-paretic and paretic limbs [NP-S1 vs. NP-S15 (*Z* *=* *−3.16, p* *<* *0.01*); P-S1 vs. P-S15 (*Z* *=* *−3.87, p* *<* *0.01*)] was observed.

During the slip training, the aborted stepping strategy reduced from 46.7% to 6.7% [fall with aborted stepping (FallAs): 23.3% to 0%; BLOBAs: 23.3% to 6.7%] for the non-paretic slip, and it reduced from 10% to 0% (FallAs: 6.7% to 0%; BLOBAs: 3.3% to 0%) for the paretic slip. The recovery stepping strategy reduced from 53.3% to 13.3% for the non-paretic slip [fall with recovery stepping (FallRs): 10% to 0%; BLOBRs:43.3% to 13.3%], and it reduced from 83.4% to 43.3% for the paretic slip (FallRs: 46.7% to 3.3%; BLOBRs:36.7% to 40%). These strategies were replaced by no balance loss strategies of walkover (NLOBWO) and skateover (NLOBSkO). These percentage distributions are presented in Figure 4B.

#### Pre-slip CoM state stability

**Figure 5:**
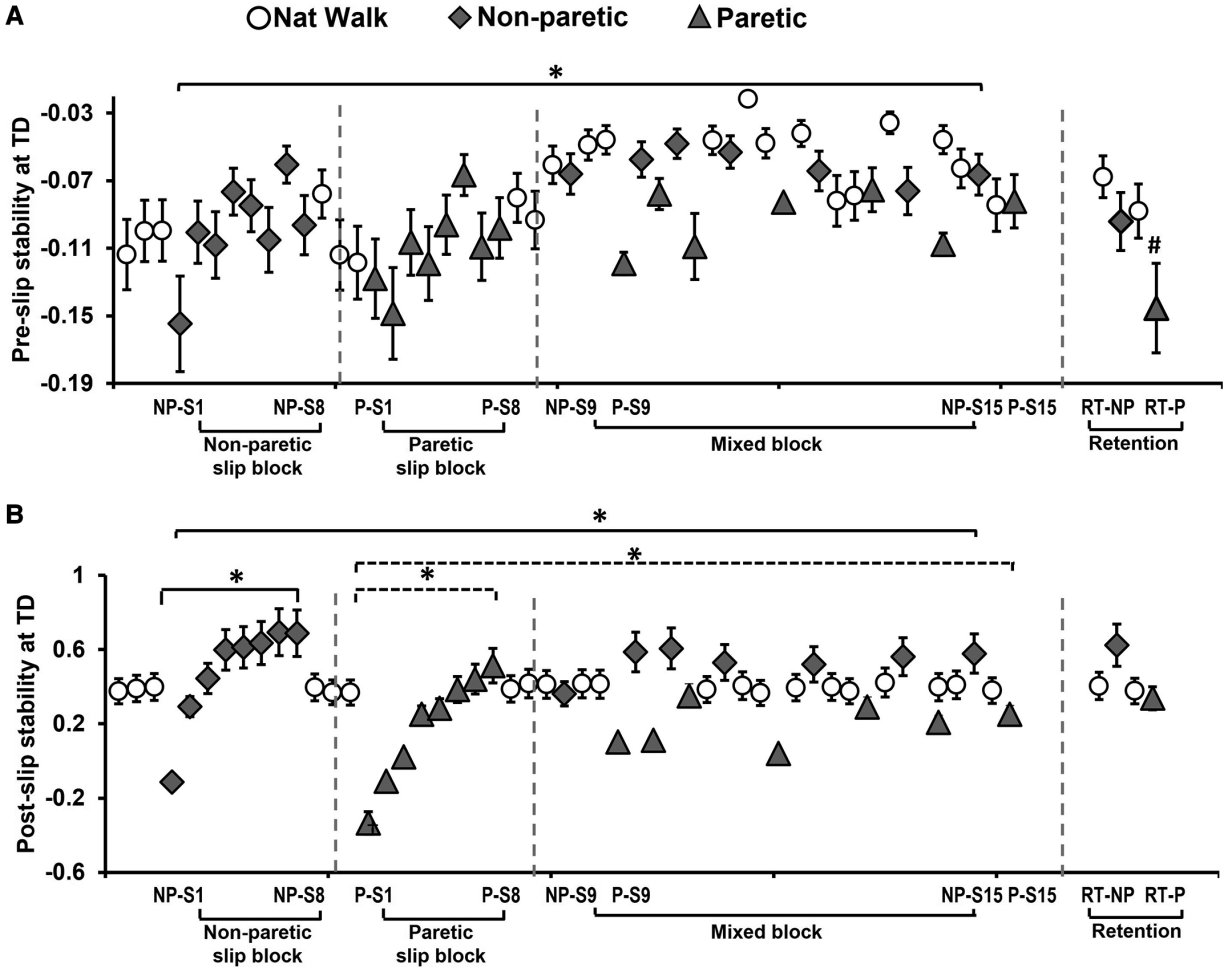
Means and standard deviations for (**A**) pre-slip stability obtained at the instance of slipping limb touchdown (TD) and (**B**) post-slip stability obtained at the instance of recovery step TD, of the training group on the non-paretic (NP) and paretic (P) slip trials. Significant differences (*p* < 0.01) are indicated by *. Significant differences between retention slips with their corresponding last training non-paretic (NP-S15) and paretic slip (P-S15) (*p* < 0.05) are indicated by ^#^. Filled diamond symbols indicate non-paretic slip trials, triangular symbols indicate paretic slip trials and open circular symbols indicate natural walking trials. Solid lines indicate differences in non-paretic slips while dotted lines indicate differences in paretic slips.

The one-way ANOVA revealed a significant main effect of trial on pre-slip CoM state stability *[F* (*29,841*) *=* *2.07, p* *<* *0.001]* (Figure 5A). Planned comparisons between NP-S1 vs. NP-S8 *[t* (*29*) *=* *−2, p* *>* *0.01]* and P-S1 vs. P-S8 *[t* (*29*) *=* *−1.7, p* *>* *0.01]* demonstrated no significant improvement in pre-slip stability at TD with non-paretic and paretic slips. There was no significant difference in pre-slip stability within the mixed block {NP-S9 vs. NP-S15 *[t* (*29*) *=* *0.01, p* *>* *0.01]*, P-S9 vs. P-S15 *[t* (*29*) *=* *−1.09, p* *>* *0.01]*}. However, a significant improvement in pre-slip stability was noted in the last training trial when compared to the first slip on the non-paretic limb [NP-S1 vs. NP-S15 *[t* (*29*) *=* *−2.78, p* *<* *0.01]*. There was no significant difference in pre-slip stability on comparing the first slip on the paretic limb [P-S1 vs. P-S15 *[t* (*29*) *=* *−2.17, p* *>* *0.01]*.

#### Post-slip CoM state stability

The one-way ANOVA revealed a significant main effect of trial on post -slip CoM state stability *[F* (*29,841*) *=* *15.98, p* *<* *0.001]* (Figure 5B). Planned comparisons between NP-S1 vs. NP-S8 *[t* (*29*) *=* *−9.43, p* *<* *0.01]* and P-S1 vs. P-S8 *[t* (*29*) *=* *−9.31, p* *<* *0.01]* demonstrated significant improvement in post-slip stability at TD with block slip training. There was no significant improvement within the mixed block for post-slip stability on both non-paretic slips {NP-S9 vs. NP-S15 *[t* (*29*) *=* *−2.36, p* *>* *0.01]*} and paretic slips {P-S9 vs. P-S15 *[t* (*29*) *=* *−2.45, p* *>* *0.01]*}. However, there was a significant improvement in stability on the last training trial when compared to the first slip on both non-paretic and paretic limb [NP-S1 vs. NP-S15 *[t* (*29*) *=* *−9.381, p* *<* *0.01],* P-S1 vs. P-S15 *[t* (*29*) *=* *−7.02, p* *<* *0.01]*].

#### Maximum slip displacement

**Figure 6:**
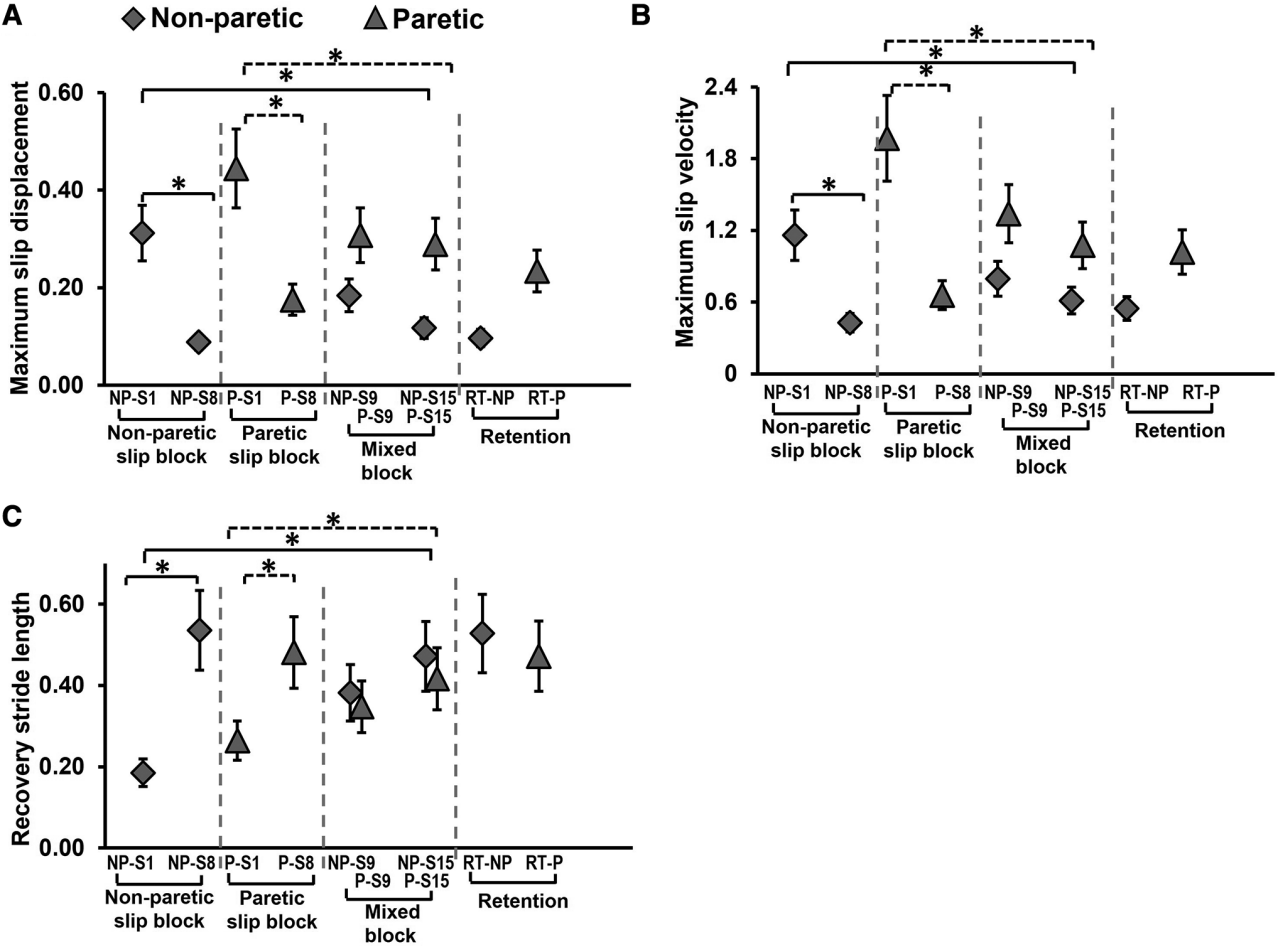
Means and standard deviations for (**A**) maximum slip displacement, (**B**) maximum slip velocity, and (**C**) recovery stride length of the training group on the non-paretic (NP) and paretic (P) slip trials. Significant differences (*p* < 0.01) are indicated by *. Filled diamond symbols indicate non-paretic slip trials and triangular symbols indicate paretic slip trials. Solid lines indicate differences in non-paretic slips while dotted lines indicate differences in paretic slips.

There was a significant main effect of trial on slip displacement [*F* (*7,189*) *=* *20.62, p* *<* *0.001*] (Figure 6A). Planned comparisons between NP-S1 vs. NP-S8 *[t* (*29*) *=* *7.48, p* *<* *0.01]* and P-S1 vs. P-S8 *[t* (*29*) *=* *8.73, p* *<* *0.01]* demonstrated significant decrease in maximum slip displacement with block training. There was no significant decrease in slip displacement within the mixed block on non-paretic slips {NP-S9 vs. NP-S15 *[t* (*29*) *=* *2.49, p* *>* *0.01]*} and paretic slips {P-S9 vs. P-S15 *[t* (*29*) *=* *0.84, p* *>* *0.01]*}*.* However, there was a significant improvement (decrease) in slip displacement on the last training trial when compared to the first slip on both non-paretic and paretic limb {NP-S1 vs. NP-S15 *[t* (*29*) *=* *5.96, p* *<* *0.01],* P-S1 vs. P-S15 *[t* (*29*) *=* *4.73, p* *<* *0.01]*}.

#### Maximum slip velocity

There was a significant main effect of trial on slip velocity [*F* (*7,189*) *=* *33.38, p* *<* *0.001*] (Figure 6B). Planned comparisons between NP-S1 vs. NP-S8 *[t* (*29*) *=* *8.55, p* *<* *0.01]* and P-S1 vs. P-S8 *[t* (*29*) *=* *13.7, p* *<* *0.01]* demonstrated significant decrease in maximum slip velocity with block training. There was no significant decrease in slip displacement within the mixed block on non-paretic slips {NP-S9 vs. NP-S15 *[t (29)* *=* *2.15, p* *>* *0.01]*} and paretic slips {P-S9 vs. P-S15 *[t (29)* *=* *2.66, p* *>* *0.01]*}*.* However there was a significant improvement (decrease) in slip velocity on the last training trial when compared to the first slip on both the non-paretic and paretic limb {NP-S1 vs. NP-S15 *[t (29)* *=* *6.4, p* *<* *0.01]*, P-S1 vs. P-S15 *[t (29)* *=* *8.43, p* *<* *0.01]*}.

#### Recovery stride length

There was a significant main effect of trial on recovery stride length [*F (7,189)* *=* *16.27, p* *<* *0.001*] (Figure 6C). Planned comparisons between NP-S1 vs. NP-S8 *[t (29)* *=* *−9.28, p* *<* *0.01]* and P-S1 vs. P-S8 *[t (29)* *=* *−5.58, p* *<* *0.01]* demonstrated significant increase in stride length with block training. There was no significant change in stride length within the mixed block on non-paretic slips {NP-S9 vs. NP-S15 *[t (29)* *=* *−2.03, p* *>* *0.01]*} and paretic slips {P-S9 vs. P-S15 *[t (29)* *=* *−2.73, p* *>* *0.01]*}*.* However, there was a significant improvement (increase) in stride length on the last training trial when compared to the first slip on both non-paretic and paretic limb {NP-S1 vs. NP-S15 *[t (29)* *=* *−5.74, p* *<* *0.01,* P-S1 vs. P-S15 *[t (29)* *=* *−3.64, p* *<* *0.01]*}.

#### Kinematic contributors of stability

Stepwise linear regression results showed that pre-slip stability was determined by the step length, knee angle of the slipping limb at pre-slip TD, and regular gait speed (*r* = 0.54, *p* < 0.05) (Table 2). Among these factors, step length (partial *r*^2^ = 0.24 in Table 2) contributed most to the changes in pre-slip stability. For post-slip stability, the analysis suggested that the slip distance at recovery step touchdown could attribute to over 50% of the changes during repeated slip training (*r* = 0.72, *p* < 0.05). Our results further revealed that slip distance was determined by pre-slip stability, knee angle, ankle angle, and trunk angle at recovery step touchdown (*r* = 0.45, *p* < 0.05), and knee angle at recovery limb touch down is the key factor affecting the changes in slip distance (partial *r*^2^ = 0.16 in Table 2).

**Table 2:** The stepwise regression and partial correlation results for pre-slip stability, post-slip stability, and slip distance at recovery step touchdown. STD: slipping limb touchdown, RTD: recovery limb touchdown.

Outcome	Stepwise regression				
	Predictor	*p*-value	Beta	*r* value	partial *r*^2^
Pre-slip stability	Knee angle at STD	0.003	−0.001	0.54	0.02
	Step length	<0.001	−0.76		0.24
	Gait speed	<0.001	−0.03		0.03
Post-slip stability	Slip distance	<0.001	−2.68	0.72	0.52
Slip distance	Knee angle at RTD	<0.001	−0.002	0.45	0.16
	Ankle angle at RTD	<0.001	0.002		0.09
	Trunk angle at RTD	<0.001	0.004		0.02
	Pre-slip stability	<0.001	−0.18		0.03

### Effects of immediate retention of acquired adaptation (within training group)

#### Slip outcomes

Within-group comparison of fall outcomes for the training group to determine retention of acquired adaptation revealed there was no significant difference in fall outcomes between NP-S15 and RT-NP (*Z* *=* *0.0, p* *>* *0.01)* and between P-S15 and RT-P (*Z* *=* *0.0, p* *>* *0.01)* (Figure 4A).

#### Pre-slip CoM state stability

Within-group comparison of pre-slip stability of the training group to determine retention of acquired adaptation, showed no significant change in pre-slip stability for non-paretic slips *[t (29)* *=* *1.48, p* *>* *0.01] (*NP-S15 vs. RT-NP)*,* however, there was a significant decrease in stability for the paretic slips *[t (29)* *=* *2.37, p* *<* *0.01] (*P-S15 vs. RT-P) (Figure 5A).

#### Post-slip CoM state stability

Within-group comparison of post-slip stability of the training group to determine retention of acquired adaptation, showed no significant change in post-slip stability for the non-paretic slips *[t (29)* *=* *−7.1, p* *>* *0.01]* (NP-S15 vs. RT-NP) and paretic slips *[t (29)* *=* *−1.08, p* *>* *0.01] (*P-S15 vs. RT-P) (Figure 5B).

### Effect of slip training (between groups)

#### Slip outcomes

**Figure 7:**
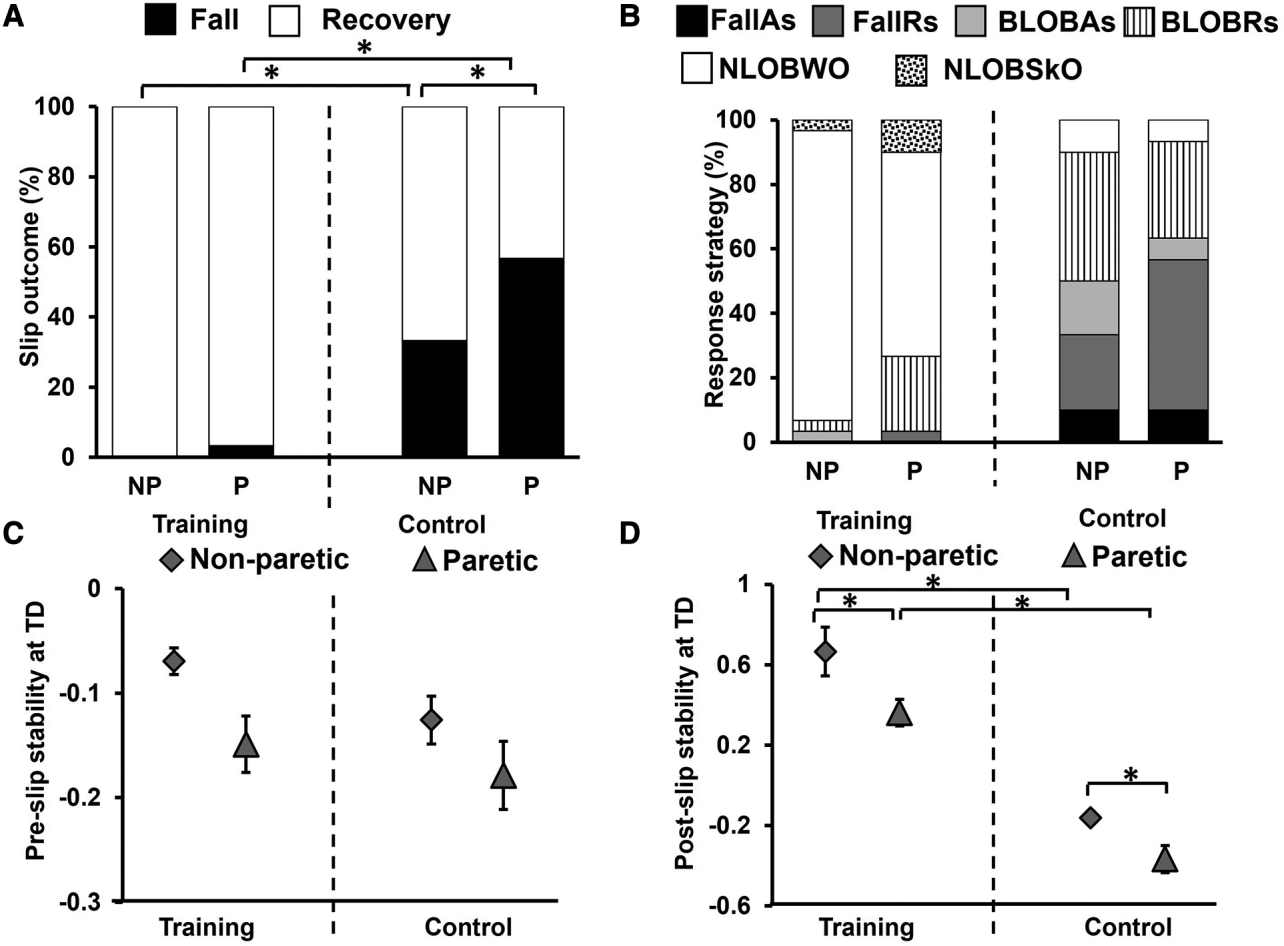
Percentage of (**A**) slip outcomes [falls and recovery (no falls)] and (**B**) response strategies employed by the training and control group on retention non-paretic (NP) and paretic (P) slip trials [fall with aborted step (FallAs), fall with recovery step (FallRs), backward loss of balance with aborted step (BLOBAs), backward loss of balance with recovery step (BLOBRs), no loss of balance walkover (NLOBWO) and no loss of balance skate over (NLOBSkO)]. Figure also shows means and standard deviations for (**C**) pre-slip stability obtained at the instance of slipping limb touchdown (TD), and (**D**) post-slip stability obtained at the instance of recovery step TD. Significant differences (*p* < 0.01) are indicated by *. Filled diamond symbols indicate non-paretic slip trials and triangular symbols indicate paretic slip trials.

The Generalized Estimating Equations for fall incidence demonstrated a significant main effect of the group (*R^2^* *=* *10.86, p* *<* *0.05*), and no main effect of the slipping limb (*R^2^* *=* *0.345, p* *>* *0.05*). In the training group compared to the control group, fall incidence was significantly lower for non-paretic slips *[U* *=* *600, p* *<* *0.01]* (training: no falls; control: 33%) and paretic slips *[U* *=* *690, p* *<* *0.01]* (training: 3%; control: 57%) (Figure 7A). Within-group analysis showed no difference in the percentage of falls between the non-paretic and paretic slips for the training group (*Z* *=* *1.0, p* *>* *0.01*), however, a significant difference was seen for the control group (*Z* *=* *2.65, p* *<* *0.01*). The control group also had greater BLOB outcomes (training: 7%; control: 57%) whereas the training group had a majority of NLOB outcomes (training: 93%; control: 10%) (Figure 7B). Specifically, our descriptive statistics on recovery strategies on the immediate retention non-paretic slip, showed that compared to the control group, the training group had a lower percent of aborted stepping strategy (FallAs: 0% and BLOBAs: 3.3% in training, FallAs: 10% and BLOBAs:16.7% in control) and a lower percent of recovery stepping strategy (FallRs: 0% and BLOBRs: 3.3% in training, FallRs: 23.3% and BLOBRs: 40% in control). Similarly, on the immediate retention paretic slip, compared to the control group the training group also showed a lower percent of aborted stepping strategy (FallAs: 0% and BLOBAs:0% in training, FallAs: 10% and BLOBAs:6.7% in control) and a lower percent of recovery stepping strategy (FallRs: 3.3% and BLOBRs:23.3% in training, FallRs: 46.7% and BLOBRs:30% in control).

#### Pre-slip CoM state stability

Between groups comparison using the 2 × 2 repeated measures ANOVA revealed a significant main effect of slipping limb [*F (1,58)* *=* *7.30, p* *<* *0.05]*; however, there was no significant group effect [*F (1,58)* *=* *2.81, p* *>* *0.05]* or significant slipping limb by group effect [*F (1,58)* *=* *0.64, p* *>* *0.05]* observed (Figure 7C). The training group showed no significant difference in pre-slip stability between retention trials on the non-paretic and paretic slip *[t (29)* *=* *2.22, p* *>* *0.01]*. Similarly, the control group showed no difference between the non-paretic and paretic limbs *[t (29)* *=* *2.06, p* *>* *0.01]*.

#### Post-slip CoM state stability

Between groups comparison using the 2 × 2 repeated measures ANOVA showed a significant main effect of slipping limb [*F (1,58)* *=* *18.57, p* *<* *0.05]* and the main effect of group in post-slip stability at TD [*F (1,58)* *=* *92.24, p* *<* *0.05]*, however, there was no slipping limb by group interaction [*F (1,58)* *=* *0.47, p* *>* *0.05]* (Figure 7D). The between-group comparison demonstrated significantly lower post-slip stability for the control group on both the non-paretic limb *[t (58)* *=* *9.31, p* *<* *0.01]* and paretic limbs *[t (58)* *=* *6.6, p* *<* *0.01]*. The training group showed a significant difference in post-slip stability between retention trials on the non-paretic and paretic slip *[t (29)* *=* *3.24, p* *<* *0.01]*. Similarly, the control group showed a significant difference between the non-paretic and paretic slips *[t (29)* *=* *2.84, p* *<* *0.01]*.

## Discussion

The study findings indicate the intact ability of community-dwelling PwCS to demonstrate training-induced behavioral adaptation and immediate retention of acquired adaptation following overground block-and-mixed perturbation-based training. This is evidenced by the significant reduction in number of falls following non-paretic and paretic slip blocks (Figure 4A). In our study, PwCS demonstrated an increase in pre-slip CoM state stability (also referred to as pre-slip stability or proactive stability) from the first to last trial of non-paretic slips only (NP-S1 to NP-S15) with no changes observed within each block or during the mixed block (Figure 5A). Further, PwCS demonstrated a significant increase in post-slip CoM state stability (also referred to as post-slip stability or reactive stability) for both non-paretic and paretic slips from the first to last trials (NP-S1 to NP-S15 and P-S1 to P-S15) and within non-paretic and paretic blocks (NP-S1 to NP-S8 and P-S1 to P-S8). However, during the mixed block training, a steady state was reached with no further change in post-slip CoM state stability for both non-paretic and paretic slips (Figure 5B). This increase in post-slip stability was accompanied by a reduction in post-slip kinematics (slip displacement and velocity) and an increase in recovery stride length in both the non-paretic and paretic slips blocks (Figure 6), affecting recovery strategy outcomes (Figure 4B). There was a decrease in backward balance losses both for the aborted stepping and recovery stepping strategies, which were replaced by no balance loss strategies of walkover and skateover similar that to see previously with healthy adults (54).

Contrary to that seen in slip-perturbation training studies in healthy older adults (showing improvement in both pre- and post-slip stability) (14, 18, 54), PwCS were not able to modulate pre-slip stability effectively, especially on the paretic side. This might potentially be attributed to the severity of stroke impairment of our participants which might limit their ability to make required modifications in (57–59) proactive strategies (60) despite being community-dwelling ambulatory adults. Our findings are in alignment with previous results in people post-stroke demonstrating impaired proactive control in the form of delayed anticipatory postural adjustments, and inability to modulate task-specific appropriate responses thereby causing incoordination of the postural muscles during voluntary movement (61–64). Further, in our study, an increase in pre-slip stability was associated with alterations in step length (shorter step) and knee angle (increased flexion) at touchdown of the slipping limb along with gait speed. Such proactive adjustments in slipping limb joint angles and spatial gait parameters are known to alter braking impulse and result in a significant reduction of slip distance, thereby reducing slip intensity (17, 54, 56). Thus, although participants in our study potentially had the knowledge of slip occurrence (expectancy) and were aware of the possible locations (either limb or spatial predictability), they probably were not able to utilize anticipatory control to make adequate proactive changes, which in turn would increase demands on reactive strategies for a successful recovery from the slip perturbations, especially on the paretic limb.

Our findings demonstrate that PwCS were able to demonstrate training-induced enhancements in reactive balance control to improve recovery outcomes. The improvement in post-slip stability was associated with reductions in slip displacement that could be attributed to changes in the post-slip ankle, knee, and trunk angle of the slipping limb. These findings are supported by our previous study in older adults that examined kinematic and kinetic factors associated with slip recovery outcomes associated with repeated slip exposure (56). Specifically, the study found that the sufficient ankle plantar flexor, knee flexor and hip extensor joint moments in early post-slip (reactive phase) are necessary to control the slip displacement and velocity, thus maintaining stability and altering recovery outcomes (56). Thus, our results align with previous slip-perturbation training studies in older adults indicating that such a training paradigm can improve reactive balance control by altering kinematic and kinetic parameters.

However, PwCS demonstrated lower post-slip stability on the paretic side slips than the non-paretic side slips during the mixed block (Figure 5B). Even though for paretic slips a recovery stepping strategy is predominately seen due to intact ability of the non-paretic (recovery limb) to step, the lower stability values on paretic slips could result from an inability of the paretic side to provide sufficient reactive vertical limb support and decreased ability to modulate the slip intensity (inability to reduce the maximum heel displacement). For the non-paretic slips, there could be a better modulation of the slip intensity by the slipping limb and the aborted strategy predominantly could be a strategy used given the motor impairment of the paretic (recovery limb) to re-establishing/and or maintaining double support phase rapidly providing vertical limb support and allowing the non-paretic limb to reactively control the slip intensity. Further, the enhanced pre-slip stability on the non-paretic side could have assisted with the greater improvements in post-slip stability. Collectively, these results indicate possible differences in adaptation between the affected and less-affected sides post-stroke and the potential need for a higher training dosage for the paretic side. From a mechanistic perspective, it is postulated that perturbation induced, trial-error based implicit training improves feedforward control of stability and limb support by predicting the upcoming context, based on the recent perturbation history (34). Improved feedforward control could prepare and assist the *post-perturbation recovery response* when a context experienced is similar to that predicted by the CNS (65). However, adaptation within the reactive system is induced possibly by parameterization of the motor response based on the available sensorimotor information to update stability and limb support control for the current context (66). The motor response is subsequently refined with practice (repeated perturbations) to maintain optimal stability and limb support and recovery states across different environments (67). However, these postulations need further validation in PwCS.

Lastly, the training group demonstrated a significant effect of the slip intervention evidenced by significantly lower falls on both the non-paretic and paretic slip trials (Figure 7A) and greater post-slip stability (Figure 7D) compared to the respective trials of the control group. Further, in the training group, both non-paretic and paretic limbs demonstrated significant retention of the improved slip outcomes (reduced falls and balance losses) and post-slip stability after a 30-minute break (NP-S15 and P-S15) (Figures 4A, 5B). The improvement seen in pre-slip stability on the non-paretic side was maintained on the retention trial (NP-S15 vs. RT-NP) (Figure 5A). The results indicate that the acquired adaptation was resistant to the 30-minute washout period and could be maintained at least on a shorter interval, demonstrating short-term retention (67). While our study focusses only on short-term retention after 30-minute washout, previous studies have demonstrated long-term improvements such as retention of acquired adaptive skills from a *single* repeated-slip training session for several months up to a year (13, 17, 54). These promising changes could be attributed to incorporation of random practice (contextual interference) (68) and overlearning (continued task practice after reaching a success criterion) (69) among healthy adults (17, 54). While the acquired adaptation in PwCS exhibiting unilateral impairment were able to resist 30-minute washout showing comparable performance to the last training trials on the non-paretic side, there was a greater motor memory decay on the paretic side. Within the CNS, retention is accomplished through the formation of new synapses and increased excitability of the neurons (70). Such consolidation is also believed to facilitate efficient retrieval of the learned motor responses (71) and can be initiated within minutes or hours (72). This postulation is supported by preliminary evidence from healthy young adults demonstrating changes in functional brain activation after a 3-day treadmill-based progressive slip perturbation training during an imagined slip condition (73). Results from this study showed significant training-induced behavioral improvement in post-slip stability and post-training increase in engagement/activation of *bilateral* dorsolateral prefrontal cortex, superior parietal lobule, inferior occipital gyrus, and lingual gyrus. It is possible that perturbation training could induce such neuroplastic changes within the brain. However, further research is needed to validate neural correlates of perturbation-induced adaptation and retention in PwCS.

Our results could have several clinical implications. First, our paradigm focused on bilateral gait-slip perturbation training as evidence indicates that training both non-paretic and paretic limbs is crucial for slip-related fall prevention (32). However, from a training protocol perspective, we chose to focus on the non-paretic slip training block to occur before the paretic training block for the following reasons. Based on our previous work on inter-limb transfer, non-paretic block training could serve as a primer to improve the ability of the paretic limb to acquire adaptations (74). Also, initiating slip training on the non-paretic limb might be clinically more feasible and ensure greater patient safety compared to training on the paretic limb first. It is possible that initiating paretic limb training could show lower tolerance to such training due to neuromuscular impairment and risk of injury on the paretic limb. This non-paretic slip training might also improve the balance confidence of PwCS and reduce their anticipation or fear of falling on encountering a subsequent block of paretic slips. Second, the “block-and-mixed” training paradigm has the potential to show greater long-term effectiveness, as the initial block training helps in facilitating motor acquisition while the later mixed (random) practice might help in better retention of acquired fall-resisting skills and improve response to unpredictable perturbations encountered in real life. Third, our findings suggest the need for future perturbation-based training protocols to incorporate greater training dosage (number of slip trials) for the paretic limb and use of washout walking trials.

Our study also has a few limitations. First, while our training protocol incorporated temporal unpredictability (exact time of slip occurrence unknown) and spatial unpredictability (limb of perturbation unknown), there is a possibility that participants might have anticipated the upcoming perturbation. This might limit the generalization of training-induced adaptive changes to real-life perturbations. However, previous evidence (33, 75, 76) indicates that although knowledge of perturbation might result in anticipatory changes in gait pattern and pre-slip stability, these changes are not enough to prevent falls. Further, adaptations in pre-slip stability were lower than in post-slip stability (Figures 5, 7C,D), thus minimizing the concern for this limitation. Second, this study only examined immediate retention effects and did not examine the generalization of findings to daily living. Future studies should examine the dose-response relationships to induce long-term retention of acquired adaptation and generalization effects on real-life falls among PwCS following such training. Third, as this study included high-functioning PwCS, the findings of this study might have limited application in individuals with acute/sub-acute stroke or those with low functioning levels. Fourth, the assessor and training personnel in our study were not blinded which might lead to bias. Lastly, our training utilizes a custom-designed overground perturbation system. This might limit the ecological validity of such a training protocol due to the lack of portability and the high cost of such a perturbation system.

## Conclusion

PwCS demonstrate the ability to acquire and retain adaptations predominantly in post-slip stability control after bilateral overground gait-slip-perturbation training under expected but unpredictable conditions to improve recovery outcomes. Given that greater adaptive changes were noted during the retention trial on the non-paretic limb, a higher dosage might be needed for paretic limb slips compared to non-paretic limb slips. Further, our findings lay the foundation to motivate the development of feasible and effective clinical rehabilitation programs focusing on training both limbs to reduce overall fall risk in PwCS.

## Data Availability

The raw data supporting the conclusions of this article will be made available by the authors, without undue reservation.
